# Self-retained cryopreserved amniotic membrane for treating severe corneal ulcers: a comparative, retrospective control study

**DOI:** 10.1038/s41598-020-73672-2

**Published:** 2020-10-12

**Authors:** Han Y. Yin, Anny M. S. Cheng, Sean Tighe, Philip Kurochkin, Jamie Nord, Swetha Dhanireddy, Robert Swan, Samuel Alpert

**Affiliations:** 1 Department of Ophthalmology and Visual Sciences, SUNY Upstate Medical University, Suit L 550 Harrison St, Syracuse, NY 13202 USA; 2 Wake Forest Baptist Eye Center, Winston-Salem, NC USA; 3 Florida International University College of Medicine, 11200 SW, 8th St, Miami, FL 33199 USA; 4 Department of Surgery, Miller School of Medicine, University of Medicine, Miami, FL USA; 5 Department of Biochemistry and Molecular Biology, Miller School of Medicine, University of Miami, Miami, FL USA; 6 TissueTech, 7300 corporate Center Drive, Suite 700, Miami, FL 33126 USA

**Keywords:** Antimicrobial responses, Infection, Bacterial infection, Corneal diseases

## Abstract

To compare the effectiveness of self- retained cryopreserved AM as an adjuvant therapy for infectious corneal ulcers. Retrospective, case–control study of 24 eyes of 24 consecutive patients with central and paracentral corneal infectious ulcers and initial visual acuity worse than 20/200. Among them, 11 eyes of 11 patients received additional placement of self-retained cryopreserved AM. Epithelialization and Best Corrected Snellen Visual Acuity (BCSVA) were compared between the two groups. At baseline, both groups had comparable age, gender, visual acuity (VA), size and location of corneal ulcer. Patients receiving additional placement of cryopreserved AM had significantly faster epithelialization within 3.56 ± 1.78 weeks vs 5.87 ± 2.20 weeks (*p* = 0.01) and achieved complete epithelialization in significantly more patients (72.7% vs 23.1% *p* = 0.04) despite overall larger baseline defect size (32.7 ± 19.5 mm^2^ vs 21.5 ± 10.7 mm^2^, *p* = 0.11). Consequently, the AM group had clinically significant BCSVA (> 3 lines) (81.8% vs 38.4%, *p* = 0.047) and total VA improvement (log MAR 0.7 ± 0.6 vs 1.6 ± 0.9, *p* = 0.016) compared to the control group at the time of complete epithelialization. In-office sutureless AM may be an effective adjuvant therapy in treating sight-threatening infectious corneal ulcers by promoting faster corneal epithelialization and overall better recovery of the VA.

## Introduction

The corneal epithelium acts as the first line of immunological defense and plays an important role in serving the optical interphase with the tear film. When this protective barrier is subjected to endogenous (e.g., severe inflammation) or exogenous (e.g., mechanical) insults, this may cause punctate erosion, which eventually evolves into a corneal epithelial defect^1^. If not treated appropriately and promptly, the persistent corneal defect can progress to severe vision-threatening complications such as infectious corneal ulcer, perforation, and endophthalmitis.

Early diagnosis and treatment of the underlying cause of the corneal ulcer are essential in restoring the ocular surface health. For infectious cases, proper topical or systemic anti-microbial therapies are required along with discontinuation of other inappropriate medications, especially topical medications with preservatives that may cause medicamentosa, i.e., ocular toxicity. To further support epithelial wound healing, adjunctive measures are implemented such as tarsorrhaphy, bandage contact lens, and amniotic membrane (AM) transplantation. Of these choices, AM not only delivers steroid-sparing anti-inflammatory, anti-scarring, and pro-regenerative properties^2,3^ but also provides a mechanical protective barrier effect. Suture of cryopreserved AM in the operating room^4–6^ or placement of self-retained cryopreserved AM in-office^7–10^ has successfully treated corneal ulcers of various etiologies. However, comparative analysis between self-retained AM and a control group has not been performed. Herein, we report the first case-controlled study to compare treatment for central and paracentral corneal infectious ulcers with the standard of care alone with and without additional placement of self-retained cryopreserved AM.

## Materials and methods

This study was approved by the SUNY Upstate State Medical University Institutional Review Boards [1593072-1]. Written informed consent was obtained in advance from all patients in accordance with the principles expressed in the Declaration of Helsinki for human subjects. A retrospective chart review was performed on consecutive patients with severe central or paracentral corneal infectious ulcers of the size of > 4mm^2^ and vision worse than 20/200 and were treated between 2016 and 2020 at SUNY Upstate Medical University. Any patient with lagophthalmos, eyelid abnormality, Bell's palsy, severe ectropion, descemetocele found to be high risk for perforation, or perforated corneal ulcer were excluded from this study. Prior to 2018, amniotic membrane utilization was not adopted by SUNY Upstate. Once self retained amniotic membrane was adopted at Upstate after 2018, patients were given the choice of amniotic membrane as an adjuvant therapy.

All patients received the standard of care including corneal scraping and microbiological culture before receiving topical broad-spectrum antimicrobial antibiotics (Vancomycin 25 mg/ml and Tobramycin 15 mg/ml). Once culture was confirmed, target antimicrobial therapy was given. For bacterial keratitis, patients received oral fluoroquinolone 500 mg twice daily for 10 days. For fungal keratitis, patients received topical fortified voriconazole or amphotericin B ophthalmic drops and a 10-day course of oral Voriconazole. For viral keratitis, patients received topical Ganciclovir and 14-days of oral valacyclovir 500 mg 3 times daily. Patients in the AM group received additional placement of cryopreserved AM, i.e. PROKERA Slim (Bio-Tissue, Inc, Miami, FL), in the office under topical anesthesia with 0.5% proparacaine hydrochloride eye drops. Briefly, cryopreserved AM was rinsed with a sterile balanced salt solution. Then, with the patient in a seated position, cryopreserved AM was gently inserted into superior fornix while the patient was looking down and AM centered covering the ulcerated defect after the patient resumed the orthophoric gaze. No additional fixation was used to secure the cryopreserved AM such as lateral tarsorrhaphy or external tape. Cryopreserved AM was left for at least 5 days and the membrane ring was gently removed using small tooth-forceps. Patients were followed daily for the first week, and then at least weekly thereafter.

The primary outcome measure was the proportion of patients that achieved complete corneal epithelialization, which was also determined by the absence of any fluorescein staining. Improvement of visual acuity (more than 1 line improvement or at least 3-line improvement) was also evaluated at 1 month as well at the time of complete epithelialization.

The statistical analyses were carried out using SPSS Software version 20 (IBM; Armonk, NY). Continuous data are reported as mean ± standard deviation and categorical data are reported as percentage. Data between treatment groups were compared using Fisher exact test and t-test for categorical data and continuous data, respectively. A *p* value < 0.05 was considered statistically significant.

## Results

A total of 24 eyes of 24 patients were included and consisted of 13 eyes of 13 patients receiving the standard of care alone and 11 eyes of 11 patients receiving additional placement of self-retained cryopreserved AM. The baseline demographic data and preoperative characteristics of all patients are summarized in Tables 1 and 2. Aside from an average younger age in the AM group (45.5 ± 13.9 vs 61.2 ± 21.6, *p* > 0.05), both groups had similar proportion of males vs. females (*p* = 0.21) and baseline vision (*p* = 0.63). At the baseline, the AM group had more centrally located ulcer 8 out of 11 eyes (72%) than control group 7 out of 13 (53%), however it was not statistically significant (*p* = 0.42). Additionally, the baseline corneal ulcer area of the AM group was overall larger than that of the control group although not statistically significant (32.7 ± 19.5 mm vs 21.5 ± 10.7 mm, *p* = 0.11).

**Table 1:** Demographics and Clinical Data of study population treated with conventional therapy and self-retained amniotic membrane as adjuvant therapy.

No	Sex/age (years)/ eye	Pathogens	Contact lens related	Ulcer site	Ulcer size (mm^2^)	Hypopyon (mm)	Complete epithelialization time (weeks)	Visual acuity	
								Pre	Final
1	M/42/OD	*S. aureus*	No	Paracentral	42	–	2.14	CF	20/20
2	F/53/OD	*A. flavus*	Yes	Central	27.5	1	5.14	CF	20/200
3	F/39/OS	*HSV*	No	Central	60	–	2.00	HM	20/60
4	M/20/OD	*P. aeruginosa*	Yes	Central	56	1.5	7.00	HM	20/1000
5	M/42/OD	*P. aeruginosa*	Yes	Central	63	1	5.86	HM	CF
6	M/52/OS	*P. aeruginosa*	Yes	Paracentral	12.25	0.5	3.71	HM	20/30
7	M/53/OD	*S. pneumoniae*	No	Central	16	2	3.43	LP	20/200
8	F/77/OS	*S. pneumoniae*	No	Paracentral	13.5	–	2.29	HM	20/50
9	M/52/OD	*S. pneumoniae*	no	Central	30	1	2.57	HM	20/200
10	M/28/OD	*S. pneumoniae*	Yes	Central	25	0.5	3.71	HM	20/60
11	F/33/OD	*P. aeruginosa*	Yes	Central	14	0.5	1.29	CF	20/25

**Table 2:** Demographics and Clinical Data of study population treated with conventional therapy.

No	Sex/age (years)/eye	Pathogens	Contact lens related	Ulcer site	Ulcer size area (mm^2^)	Hypopyon (mm)	Complete epithelialization time (weeks)	Visual acuity	
								Pre	Final
1	F/68/OD	*Presumed bacterial*	No	Central	12.5	1	4.71	HM	HM
2	F/27/OS	*P. aeruginosa*	yes	Central	35.75	1	3.57	LP	20/400
3	M/60/OD	*Presumed bacterial*	No	Paracentral	25	–	7.71	HM	20/400
4	F/53/OD	*P. aeruginosa*	Yes	Paracentral	12	–	5.85	HM	HM
5	F/73/OS	*Acinetobacter spp.*	No	Paracentral	12.5	–	6.00	HM	HM
6	M/71/OS	*P. aeruginosa*	No	Central	25	2.0	9.14	LP	LP
7	F/68/ OD	*Presumed bacterial*	No	Central	25	–	9.28	HM	CF
8	F/92/ OD	*C. parapsilosis*	No	Paracentral	7.5	–	5.00	20/800	20/300
9	F/87/ OS	*Presumed bacterial*	No	Central	24	–	6.00	HM	HM
10	M/29/OD	*P. aeruginosa*	Yes	Paracentral	12.5	0.5	3.14	HM	20/100
11	F/28/ OD	*P. aeruginosa*	Yes	Paracentral	12.25	–	2.71	HM	20/30
12	M/61/OS	*P. aeruginosa*	Yes	Central	35	1.5	8.42	HM	HM
13	F/78/ OD	*M. catarrhalis*	No	Central	40	–	4.85	HM	CF

For the patients receiving additional self-retained cryopreserved AM, bacterial keratitis was noted in 9 eyes, fungal keratitis in 1 eye, and herpes simplex virus keratitis in 1 eye. For patients receiving the standard of care only, cultured proven bacterial keratitis was noted in 8 eyes, fungal keratitis was confirmed in 1 eye while presumed bacterial keratitis was found in the remaining 4 eyes. The proportion of infectious cause was not statistically significant between the two groups (*p* = 0.65).

At one-month follow-up, 72.7% of the eyes in the AM group had complete epithelialization compared to 23.1% in the control group (*p* = 0.04). By 2 months, 100% of eyes were healed in AM group compared to 84.6% in the control group (*p* = 0.46). By 3 months, all ulcers were healed in both groups. Patients treated with AM showed faster epithelialization within an average 3.6 ± 1.8 weeks compared to 5.9 ± 2.2 weeks in the control group (*p* = 0.01).

At 1 month, the BCSVA was improved at least 1 line in 10 of the 11 eyes (90.9%) in the AM group compared to 6 of the 13 eyes (46.1%) in the control group receiving the standard of care (*p* = 0.03). Nine out of 11 eyes (81.8%) in the AM group achieved 3 lines or better in their BCSVA compared to 5 of the 13 eyes (38.4%) in the control group (*p* = 0.047). At the time of complete epithelialization, 11 of 11 (100%) eyes in the AM group showed at least 1-line improvement compared to 6 of the 13 eyes (46.1%) in the control group (*p* = 0.006). Nine of 11 eyes (81.8%) achieved 3 lines or better in the AM group compared to 5 out of 13 eyes (38.4%) in the control treatment group (*p* = 0.047). Vision significantly improved from pretreatment logMAR 2.3 ± 0.3 to final VA of 0.7 ± 0.6 (*p* < 0.00001) in the AM group, while VA improved from pretreatment logMAR 2.3 ± 0.3 to final VA of 1.6 ± 0.9 (*p* = 0.008) in the control group. The AM group showed more total VA improvement compared to control (log MAR 0.7 ± 0.6 vs 1.6 ± 0.9, *p* = 0.016). Neither group had worsened visual acuity and all cases of hypopyon resolved. Therefore, eyes receiving additional placement of self-retained cryopreserved AM showed faster complete epithelialization and clinically significant BCSVA improvement (1 line, > 3 lines, and total) compared to the control group. No complications were observed with the use of AM, aside from foreign body sensation.

## Discussion

The use of sutureless self-retained cryopreserved AM allows early intervention as it can be performed in the office not only to facilitate the treatment but also to reduce the overall cost. Previously, a number of reports have shown the effectiveness of single^15,16,18–22,26,27,29,32^ or multiple^15–18,20–28,30,31,33^ layers of sutured AM for infectious^5,6,15–23,25,27,28,33^ or non-infectious^15,17,18,21–26,28–32^ corneal ulcers and several reports have similarly shown the effectiveness of sutureless AM for infectious^7–10^ and non-infectious^7,12–14^ corneal ulcers. The three retrospective case series^7–10^ that evaluated self-retained AM for infectious corneal ulcers showed healing in 66–100% of cases in as little as 4 days along with reduced ocular surface inflammation and improved VA during 3–51 months of follow-up. Herein, we present the first retrospective control study to support that additional placement of sutureless self-retained cryopreserved AM in conjunction with the standard of care is effective in promoting significantly faster epithelialization and better visual acuity than that by the standard of care alone.

Taken together, the aforementioned cumulative clinical evidence is in alignment with the unique steroid-sparing anti-inflammatory, anti-scarring, and pro-regenerative properties of AM^10,11^. Although AM is known to contain multiple extracellular matrix components and growth factors that could contribute to these properties, research efforts supported by the National Institutes of Health over the last decade have led to the discovery of heavy chain 1 [derived from inter- α-trypsin inhibitor]–HA/pentraxin 3 (HC–HA/ PTX3) as the major active tissue component that is responsible for AM’s “multiple” therapeutic actions that extend to a number of cell types (reviewed in^2^). In brief, HC-HA/PTX3′s anti-inflammatory action works through modulating activated but not resting neutrophils, macrophages, and lymphocytes extending from innate to adaptive immune responses. This is in contrast to steroids which is known to manifest dose-dependent adverse event leading to immunocompromised state, inhibition of epithelialization, and risk of corneal melting.

This steroid-sparing anti-inflammatory effect is particular palatable in managing corneal ulcers which have an infection as the underlying cause, and may explain why placement of self-retained cryopreserved AM is beneficial in promoting corneal epithelial healing despite the baseline corneal ulcer area was relatively larger. In addition, HC-HA/PTX3′s anti-scarring action, demonstrated in human corneal fibroblasts by downregulating the TGF-β1 promoter activity^34^ and its anti-angiogenic action by inhibition of endothelial tube formation^35^ may help explain why the resultant visual acuity is better in the AM group despite the baseline corneal ulcers were more centrally located.

Finally, corneal ulcer disrupts the integrity of the ocular surface, which can result in further damage from the mechanical friction. The cryopreserved AM may serve as a safe mechanical barrier, preventing further fricational trauma to the ocular surface. Furthermore, various studies have reported AM to retain an antimicrobial property^36–39^. If confirmed, this may also suggest why the epithelialization occurred much faster in the AM group. Yet it remains unknown whether AM contains antimicrobial properties or simply enhances the delivery of fortified medications to the ocular surface^40,41^. Further prospective and randomized control studies could help to confirm the efficacy of self-retained cryopreserved AM and as an adjunctive treatment for infectious corneal ulcers.
